# FAM83H is involved in the progression of hepatocellular carcinoma and is regulated by MYC

**DOI:** 10.1038/s41598-017-03639-3

**Published:** 2017-06-12

**Authors:** Kyoung Min Kim, See-Hyoung Park, Jun Sang Bae, Sang Jae Noh, Guo-Zhong Tao, Jung Ryul Kim, Keun Sang Kwon, Ho Sung Park, Byung-Hyun Park, Ho Lee, Myoung Ja Chung, Woo Sung Moon, Karl G. Sylvester, Kyu Yun Jang

**Affiliations:** 1 Department of Pathology, Chonbuk National University Medical School, Research Institute of Clinical Medicine of Chonbuk National University-Biomedical Research Institute of Chonbuk National University Hospital and Research Institute for Endocrine Sciences, Jeonju, Republic of Korea; 2 Department of Bio and Chemical Engineering, Hongik University, Sejong, Republic of Korea; 3 Forensic Medicine, Chonbuk National University Medical School, Research Institute of Clinical Medicine of Chonbuk National University-Biomedical Research Institute of Chonbuk National University Hospital and Research Institute for Endocrine Sciences, Jeonju, Republic of Korea; 4 Department of Surgery, Division of Pediatric Surgery, Stanford University School of Medicine, Stanford, California, USA; 5 Orthopedic Surgery, Chonbuk National University Medical School, Research Institute of Clinical Medicine of Chonbuk National University-Biomedical Research Institute of Chonbuk National University Hospital and Research Institute for Endocrine Sciences, Jeonju, Republic of Korea; 6 Preventive Medicine, Chonbuk National University Medical School, Research Institute of Clinical Medicine of Chonbuk National University-Biomedical Research Institute of Chonbuk National University Hospital and Research Institute for Endocrine Sciences, Jeonju, Republic of Korea; 7 Biochemistry, Chonbuk National University Medical School, Research Institute of Clinical Medicine of Chonbuk National University-Biomedical Research Institute of Chonbuk National University Hospital and Research Institute for Endocrine Sciences, Jeonju, Republic of Korea

## Abstract

Recently, the roles of FAM83H in tumorigenesis have been interested and increased expression of FAM83H and MYC in hepatocellular carcinoma (HCC) have been reported. Therefore, we investigated the expression and role of FAM83H in 163 human HCCs and further investigated the relationship between FAM83H and oncogene MYC. The expression of FAM83H is elevated in liver cancer cells, and nuclear expression of FAM83H predicted shorter survival of HCC patients. In HLE and HepG2 HCC cells, knock-down of FAM83H inhibited proliferation and invasive activity of HCC cells. FAM83H induced expression of cyclin-D1, cyclin-E1, snail and MMP2 and inhibited the expression of P53 and P27. In hepatic tumor cells derived from Tet-O-MYC mice, the expression of mRNA and protein of FAM83H were dependent on MYC expression. Moreover, a chromatin immunoprecipitation assay demonstrated that MYC binds to the promotor of FAM83H and that MYC promotes the transcription of FAM83H, which was supported by the results of a dual-luciferase reporter assay. In conclusion, we present an oncogenic role of FAM83H in liver cancer, which is closely associated with the oncogene MYC. In addition, our results suggest FAM83H expression as a poor prognostic indicator of HCC patients.

## Introduction

Family with sequence similarity 83, member h (FAM83H) was identified, from a genome-wide search, as having the genetic etiology of human autosomal dominant hypocalcified amelogenesis imperfecta^1^. Thereafter, various mutations of FAM83H have been detected in amelogenesis imperfecta^1–5^ and FAM83H-associated amelogenesis imperfecta is reported to be the most prevalent form of amelogenesis imperfecta^3, 6^. Therefore, studies on FAM83H have typically been focused on tooth development. However, the cytoplasmic localization of FAM83H protein suggests that FAM83H might be involved in other cellular processes, including tumorigenesis^2, 5^. Increased expression of FAM83H in cancer tissue compared with normal tissue has been presented in recent microarray data^7^. In colorectal cancer, FAM83H contributes to the progression of cancer *via* regulating keratin cytoskeleton organization^8–10^. FAM83H overexpression along with aberrant localization of CK-1α could contribute to the progression of colorectal cancer through keratin cytoskeleton organization^8^. Another study showed that FAM83H could be an important molecule that can cause androgen independent prostate cancer progression^11^. However, although there are numerous reports of FAM83H in amelogenesis imperfecta, the studies investigating the role of FAM83H in human malignant tumors have been limited.

Hepatocellular carcinoma (HCC) is a highly frequent and lethal primary malignant neoplasm of the liver^12^ and various molecular alterations are involved in the development of HCC^13^. Among the various tumorigenic molecules, MYC is one of the most potent oncogenes of HCC^14^. MYC regulates the expression of more than 15% of all human genes and orchestrates proliferation, differentiation, self-renewal, apoptosis, and anti-tumor immunity^15–18^. Overexpression of MYC was observed in various human cancers and transgenic overexpression of MYC was sufficient to induce HCCs from murine hepatocytes^19^. Therefore, when considering the extensive transcriptional role of MYC in tumorigenesis, we predicted that MYC could also act as a transcription factor for FAM83H in part of their oncogenic roles in HCC tumorigenesis. Supportively, recent microarray analysis has shown that FAM83H gene expression is up-regulated in human HCCs^20^. Therefore, because the exact role of FAM83H in tumorigenesis has not been clear up to date, a study investigating the relationship between MYC and FAM83H in liver would be helpful in understanding the role of FAM83H in human carcinogenesis. Based on this rationale, we investigated the oncogenic role of FAM83H in HCC in conjunction with the oncogene MYC in liver cancer cells and human HCC tissue samples.

## Results

### The expression of FAM83H is higher in liver cancer cells and its expression predicts shorter survival of hepatocellular carcinoma patients

**Figure 1:**
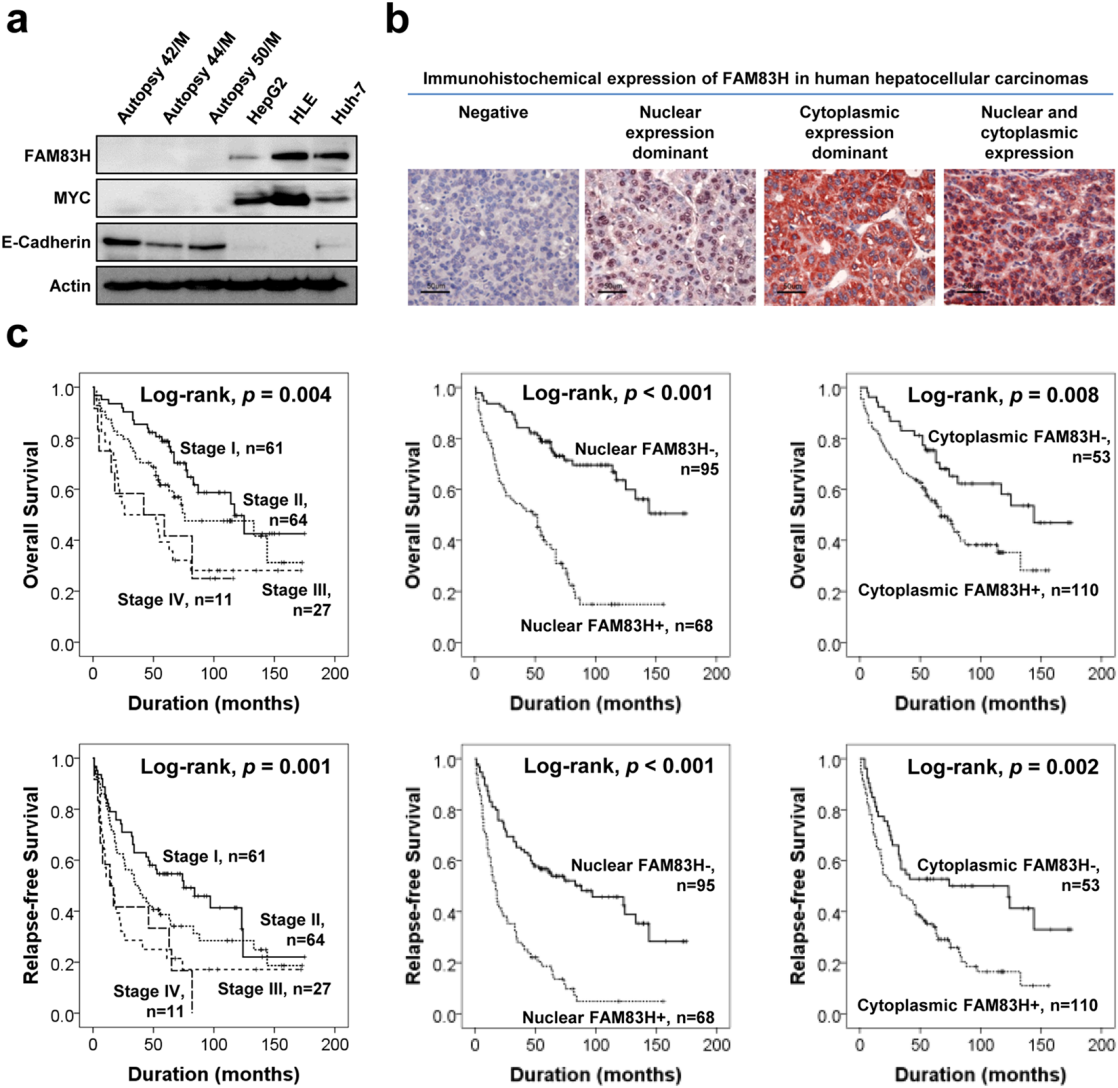
The expression and prognostic significance of FAM83H in human hepatocellular carcinomas. (**a**) Western blot analysis in normal livers from autopsy and the hepatocellular carcinoma cells lines HepG2, HLE, and Huh-7 for the FAM83H, MYC, E-cadherin, and actin. (**b**) Immunohistochemical expression of FAM83H in human hepatocellular carcinoma tissue samples. (**c**) Kaplan-Meier survival analysis according to the tumor stage, nuclear FAM83H expression, and cytoplasmic FAM83H expression for the overall survival and relapse-free survival in 163 hepatocellular carcinoma patients.

We evaluated the expression of FAM83H in normal liver and HCC cell lines. Normal liver tissue was obtained from the three autopsies; the causes of death of these individuals were not related with liver disease (two cases of head trauma [42 year-old man and 50 year-old man] and one case of ischemic heart disease [44 year-old man]) and there were no specific abnormalities in the livers observed by post-mortal liver functional tests and histologic examination. Histologic slides did not show any post-mortal degenerative changes in the livers. In normal liver tissue samples, the expression of FAM83H and MYC was undetectable and the expression of E-cadherin and actin were similar (Fig. 1a). In contrast, three HCC cell lines showed considerable expression of FAM83H and MYC (Fig. 1a). In addition, when we searched the Oncomine database for FAM83H, tissue type of liver, comparison of expression level between normal and carcinoma, and *P* value less than 0.001, two data sets matched our search criteria^21^. The expression of mRNA of FAM83H was significantly higher in liver cancer tissue compared to normal liver tissue (Supplementary Fig. S2). Thereafter, we evaluated FAM83H expression in human HCC tissue samples. As we have shown in Fig. 1b, FAM83H is expressed in both the nuclei and cytoplasm of tumor cells. Therefore, we separately evaluated the nuclear and cytoplasmic expression of FAM83H and grouped the results into negative and positive groups for nuclear- and cytoplasmic-expression of FAM83H. The cut-off points for the nuclear and cytoplasmic expression of FAM83H were seven and six, respectively (Supplementary Fig. S3). The cases with an immunohistochemical staining score of seven or greater were considered positive for nuclear FAM83H expression. Cytoplasmic FAM83H expression was considered positive if the immunohistochemical score was equal or greater than six. With these cut-off points, 42% (68/163) and 67% (110/163) of HCCs were classified as nuclear FAM83H positive and cytoplasmic FAM83H positive, respectively. Nuclear FAM83H expression was significantly associated with high preoperative serum levels of α-fetoprotein (*p* = 0.026), higher tumor stage (*p* < 0.001), tumor size (*p* = 0.046), vascular invasion (*p* < 0.001), and cytoplasmic FAM83H expression (*p* < 0.001). Cytoplasmic FAM83 expression was significantly associated with sex (*p = *0.004), HCV infection (*p* = 0.023), higher tumor stage (*p* = 0.024), and vascular invasion (*p* = 0.008) (Table 1).

**Table 1:** Clinicopathologic variables and the expression of FAM83H in 163 hepatocellular carcinomas.

Characteristics		No.	FAM83H, nuclear		FAM83H, cytoplasm	
			Positive	*p*	Positive	*p*
Sex	Male	141	61 (43%)	0.311	101 (72%)	0.004
	Female	22	7 (32%)		9 (41%)	
Age, y	<55	65	28 (43%)	0.774	39 (60%)	0.097
	≥55	98	40 (41%)		71 (72%)	
AFP, ng/mL	<100	107	38 (36%)	0.026	73 (68%)	0.781
	≥100	56	30 (54%)		37 (66%)	
HBV	Negative	43	16 (37%)	0.485	33 (77%)	0.131
	Positive	120	52 (43%)		77 (64%)	
HCV	Negative	153	64 (42%)	0.909	100 (65%)	0.023
	Positive	10	4 (40%)		10 (100%)	
Liver cirrhosis	Absence	87	38 (44%)	0.587	59 (68%)	0.923
	Presence	76	30 (39%)		51 (67%)	
Bilirubin, mg/dl	<0.7	75	31 (41%)	0.927	53 (71%)	0.423
	≥0.7	88	37 (42%)		57 (65%)	
Albumin, ng/dl	<3.5	21	8 (38%)	0.718	16 (76%)	0.362
	≥3.5	142	60 (42%)		94 (66%)	
Tumor stage	I	61	16 (26%)	<0.001	35 (57%)	0.024
	II	64	29 (45%)		47 (73%)	
	III	27	13 (48%)		17 (63%)	
	IV	11	10 (91%)		11 (100%)	
Tumor size, cm	≤5	110	40 (36%)	0.046	74 (67%)	0.934
	>5	53	28 (53%)		36 (64%)	
Vascular invasion	Absence	77	21 (27%)	<0.001	44 (57%)	0.008
	Presence	86	47 (55%)		66 (77%)	
Histologic grade	Low	102	40 (39%)	0.402	69 (68%)	0.954
	High	61	28 (46%)		61 (41%)	
FAM83H, cytoplasm	Negative	53	9 (17%)	<0.001		
	Positive	110	59 (54%)			

AFP, a-fetoprotein; HBV, hepatitis B virus; HCV, hepatitis C virus. The *p* values were calculated by Chi square test.

In univariate analysis, tumor stage (*p* = 0.006), vascular invasion (*p* < 0.001), nuclear FAM83H expression (*p* < 0.001), and cytoplasmic FAM83H expression (*p* = 0.009) were significantly associated with overall survival (OS) of HCC patients. The factors significantly associated with relapse-free survival (RFS) in the univariate analysis were preoperative serum level of α-fetoprotein (*p* = 0.007), preoperative serum level of albumin (*p* = 0.024), tumor stage (*p* = 0.002), tumor size (*p* = 0.031), vascular invasion (*p* < 0.001), nuclear FAM83H expression (*p* < 0.001), and cytoplasmic FAM83H expression (*p* = 0.002) (Table 2, Fig. 1c).

**Table 2:** Univariate and multivariate Cox regression analysis for overall survival and relapse-free survival in 163 hepatocellular carcinoma patients.

Characteristics	No.	OS		RFS	
		HR (95% CI)	*p*	HR (95% CI)	*p*
Univariate Cox regression analysis					
Sex, male (*vs* female)	141/163	0.792 (0.409–1.535)	0.489	0.807 (0.452–1.443)	0.470
Age, ≥55 (*vs* <55)	98/163	1.399 (0.887–2.207)	0.149	1.456 (0.989–2.144)	0.057
AFP ≥100 ng/ml (*vs* <100 ng/ml)	56/163	1.532 (0.990–2.370)	0.056	1.680 (1.155–2.442)	0.007
Albumin <3.5 ng/dl (*vs* ≥3.5 ng/dl)	21/163	1.713 (0.962–3.052)	0.067	1.795 (1.081–2.981)	0.024
Bilirubin, ≥0.7 mg/dl (*vs* <0.7 mg/dl)	88/163	1.295 (0.842–1.994)	0.240	1.124 (0.778–1.624)	0.534
Tumor stage, I	61/163	1	0.006	1	0.002
II	64/163	1.514 (0.888–2.583)	0.128	1.442 (0.922–2.256)	0.109
III	27/163	2.572 (1.418–4.663)	0.002	2.327 (1.366–3.963)	0.002
IV	11/163	2.706 (1.254–5.838)	0.011	2.815 (1.454–5.450)	0.002
Tumor size>5 cm (*vs* ≤5 cm)	53/163	1.414 (0.906–2.210)	0.127	1.521 (1.038–2.228)	0.031
Vascular invasion, presence (*vs* absence)	86/163	2.167 (1.385–3.390)	<0.001	2.010 (1.373–2.941)	<0.001
Histologic grade high (*vs* low)	61/163	1.456 (0.941–2.254)	0.092	1.446 (0.994–2.103)	0.054
FAM83H nuclear positive (*vs* negative)	68/163	4.020 (2.543–6.353)	<0.001	3.100 (2.117–4.540)	<0.001
FAM83H cytoplasm positive (*vs* negative)	110/163	1.946 (1.178–3.215)	0.009	1.958 (1.272–3.013)	0.002
Multivariate Cox regression analysis, Model 1^*^					
Albumin <3.5 ng/dl (*vs* ≥3.5 ng/dl)				2.233 (1.320–3.776)	0.003
Vascular invasion, presence (*vs* absence)				1.939 (1.303–2.886)	0.001
Tumor stage, I		1	0.005		
II		1.302 (0.760–2.230)	0.336		
III		2.906 (1.588–5.320)	<0.001		
IV		1.383 (0.608–3.149)	0.439		
FAM83H nuclear positive (*vs* negative)		4.202 (2.604–6.783)	<0.001	2.907 (1.979–4.272)	<0.001
Multivariate Cox regression analysis, Model 2^**^					
FAM83H cytoplasm positive (*vs* negative)		1.753 (1.058–2.902)	0.029	1.852 (1.194–2.872)	0.006

HBV, hepatitis B virus; HCV, hepatitis C virus; AFP, α-fetoprotein; HR, hazard ratio. *Variables considered in multivariate analysis Model 1 were tumor stage, tumor size, vascular invasion, α-fetoprotein level, albumin level, nuclear expression of FAM83H, and cytoplasmic expression of FAM83H. **Variables considered in multivariate analysis Model 2 were tumor stage, tumor size, vascular invasion, α-fetoprotein level, albumin level, and cytoplasmic expression of FAM83H.

The factors significantly associated with OS or RFS in univariate analysis were included in multivariate analysis. The factors included in multivariate analysis were tumor stage, tumor size, vascular invasion, α-fetoprotein level, albumin level, nuclear expression of FAM83H, and cytoplasmic expression of FAM83H. Multivariate analysis revealed tumor stage (overall *p* = 0.005) and nuclear FAM83H expression (*p* < 0.001) to be independent indicators of poor prognosis of OS of HCC patients (Table 2, Model 1). Nuclear expression of FAM83H indicated a 4.202-fold (95% confidence interval; 2.604–6.783) greater risk of death. The factors significantly associated with RFS by multivariate analysis were serum level of albumin (p = 0.003), vascular invasion (*p* = 0.001), and nuclear FAM83H expression (*p* < 0.001) (Table 2, Model 1). Nuclear expression of FAM83H predicted a 2.907-fold (95% confidence interval; 1.979–4.272) greater risk of relapse or death of HCC patients. In multivariate analysis, cytoplasmic expression of FAM83H was not found to be an independent prognostic indicator of HCC patients. This might be associated with the significant correlation between cytoplasmic and nuclear expression of FAM83H (Table 1). Therefore, when we performed multivariate analysis without including nuclear expression of FAM83H, cytoplasmic expression of FAM83H was also an independent indicator of poor prognosis of HCC patients (OS; *p *= 0.029, RFS; *p * = 0.006) (Table 2, Model 2).

### Knock-down of FAM83H inhibits proliferation and invasiveness of hepatocellular carcinoma cells

**Figure 2:**
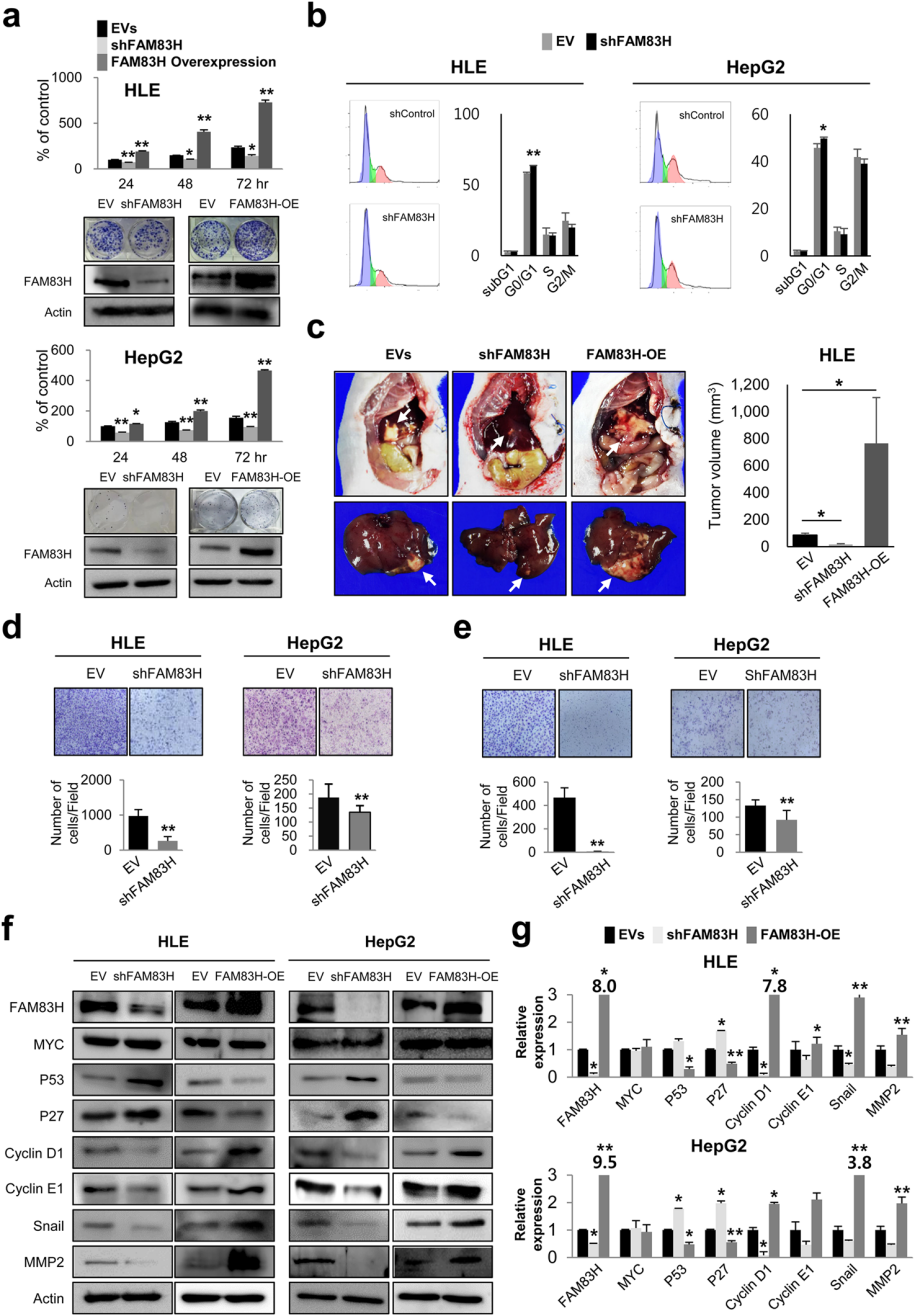
The expression of FAM83H is associated with the proliferation, cell cycle progression, and invasiveness of hepatocellular carcinoma cells. (**a**) The knock-down of FAM83H significantly inhibited the proliferation and overexpression of FAM83H induced by transfection of FAM83H increased proliferation of both HLE and HepG2 cells as indicated with MTT and colony-forming assays. (**b**) The knock-down of FAM83H by shRNA for FAM83H induced G0/G1 arrest in both HLE and HepG2 cells as indicated by flow-cytometry cell cycle analysis. (**c**) The size of tumors transplanted HLE cells in nude mice was significantly smaller with knock-down of FAM83H, and FAM83H overexpression increased tumor size in liver in nude mice. Arrows indicate hepatic tumors. (**d**,**e**) The knock-down of FAM83H significantly inhibited migration (**d**) and invasion (**e**) of both HLE and HepG2 cells. (**f,g**) The knock-down of FAM84H by shRNA for FAM83H increased expression of protein and mRNA of P53 and P27, but decreased expression of protein and mRNA of cyclin D1, cyclin E1, snail, and MMP2 in both HLE and HepG2 cells. Overexpression of FAM83H decreased expression of protein and mRNA of P53 and P27, but increased expression of protein and mRNA of cyclin D1, cyclin E1, snail, and MMP2 in both HLE and HepG2 cells. However, the expression of protein and mRNA of MYC was not changed with either knock-down or overexpression of FAM83H. EV, empty vector; EVs, co-transfection of empty vectors for shRNA and overexpression; OE, overexpression; the *p* values were calculated by Student’s *t*-test; *p < 0.05; **p < 0.001.

Because the expression of FAM83H was higher in HCC cells compared to normal liver, and the expression of FAM83H predicted shorter survival of HCC patients, we investigated the effect of the inhibition of FAM83H on the proliferation of two HCC cell lines (HLE and HepG2). The proliferation of HLE and HepG2 HCC cells were inhibited with knock-down of FAM83H and increased with the overexpression of FAM83H (Fig. 2a). Cell cycle analysis by flow cytometry revealed an increase in the G0/G1 population with a knock-down of FAM83H (Fig. 2b). In addition, the tumor size was significantly small with knock-down of FAM83H, and FAM83H overexpression increased tumor size in an orthotopic animal model (Fig. 2c). Moreover, the migration and invasive activity of HCC cells were significantly inhibited with knock-down of FAM83H (Fig. 2d,e). The expression of mRNAs and proteins involved in cell cycle progression or epithelial-mesenchymal transition (EMT) such as cyclin D1, cyclin E1, snail, and MMP2 decreased with a knock-down of FAM83H and increased with FAM83H overexpression (Fig. 2f,g). The expression of mRNA and protein of P53 and P27 were increased with knock-down of FAM83H and decreased with FAM83H overexpression. However, the expression of mRNA and protein of MYC were not significantly altered with either a knock-down or an overexpression of FAM83H (Fig. 2f,g).

### The expression of FAM83H is transcriptionally controlled by MYC

**Figure 3:**
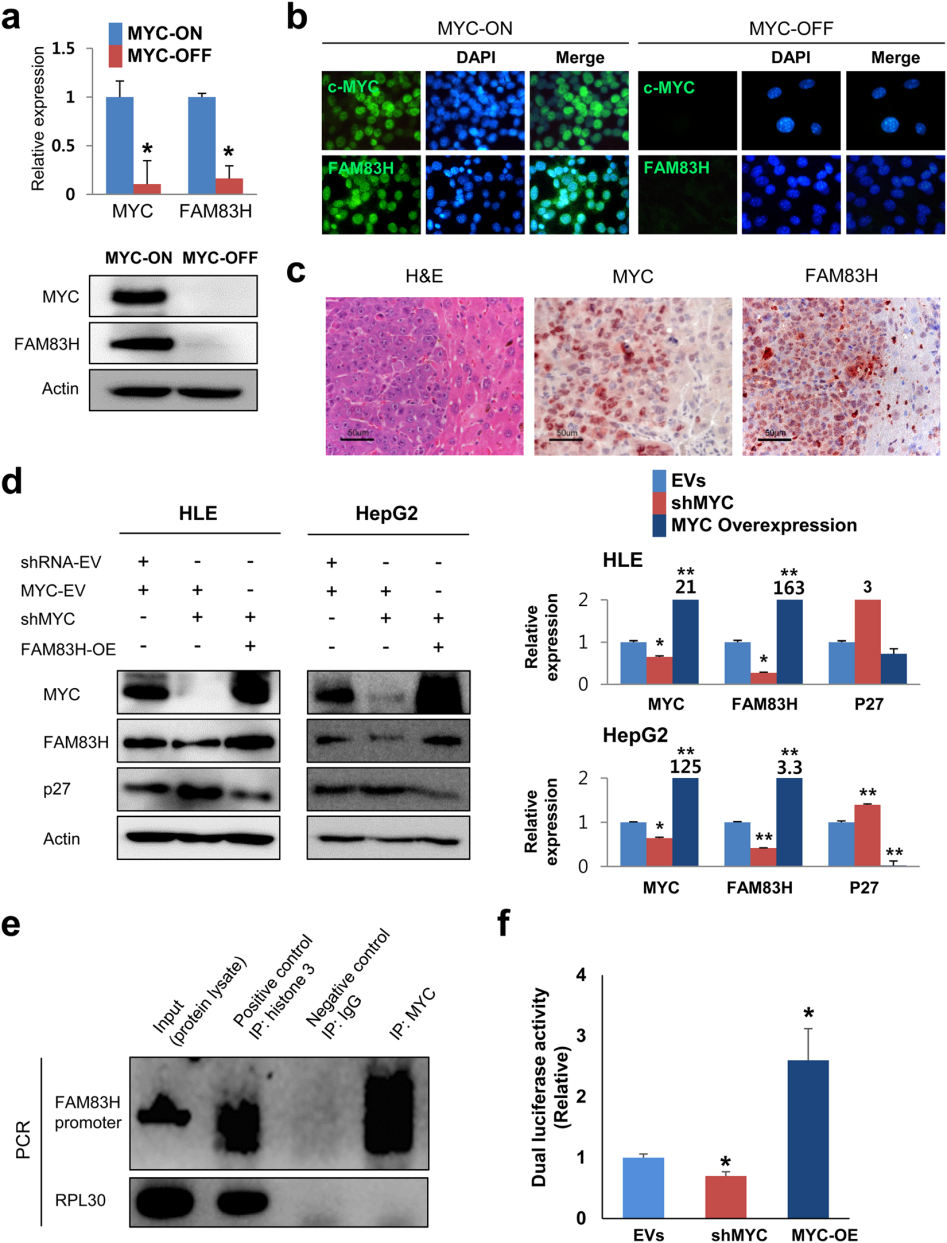
The expression of FAM83H is transcriptionally controlled by MYC. (**a**) In the cell lines derived from established liver tumors in the Tet-O-MYC mice, the expression of mRNA and protein of FAM83H decreased with inhibition of MYC by *via* 5 ng/ml doxycycline in culture media for five days. (**b**) The expression of FAM83H was decreased in MYC-OFF cells in immunofluoresence images. (**c**) Immunohistochemical expression of FAM83H is higher in MYC-expressing tumor cells compared with adjacent non-neoplastic hepatocytes in the tissue samples obtained from tumor-containing liver of Tet-O-MYC mice. (**d**) The expression of protein and mRNA of FAM83H decreased with knock-down of MYC and increased with overexpression of MYC in both HLE and HepG2 human hepatocellular carcinoma cell lines. In contrast, the expression of protein and mRNA of P27 increased with knock-down of MYC and decreased with overexpression of MYC in both HLE and HepG2 cells. (**e**) Chromatin immunoprecipitation assay shows the binding of MYC to the promotor of FAM83H. (**f**) Co-transfection of FAM83H promoter-luciferase reporter plasmid and a MYC overexpression vector significantly increased the luciferase activity. In contrast, co-transfection of FAM83H promoter-luciferase reporter plasmid and shRNA for MYC decreased the luciferase activity. EVs, co-transfection of empty vectors for shRNA and overexpression; OE, overexpression; the *p* values were calculated by Student’s *t*-test; *p < 0.05; **p < 0.001.

As we have shown in Fig. 2, the expression of MYC was not affected by the expression of FAM83H. However, the expression of both FAM83H and MYC were higher in HCC cell lines compared with normal livers. In addition, when we searched public data from the cBioPortal database^22, 23^, there was a significant correlation between the genetic alteration of FAM83H and MYC genes (Fisher Exact test, *p* < 0.001 and *p* = 0.001) (Supplementary Fig. S4). Therefore, we hypothesized that the expression of FAM83H might be dependent on the expression of MYC and evaluated the expression of FAM83H in cell lines derived from established liver tumors in Tet-O-MYC mice (Supplementary Fig. S1). As expected, the expression of mRNA and protein of FAM83H was lower when MYC was inhibited (MYC-OFF) by adding 5 ng/ml doxycycline to the culture media for five days (Fig. 3a). Immunofluoresence imaging also showed decreased expression of FAM83H in MYC-OFF cells (Fig. 3b). In addition, immunohistochemical expression of FAM83H was higher in MYC-expressing tumor cells compared with adjacent non-neoplastic hepatocytes in the tissue samples obtained from the livers of Tet-O-MYC mice (Fig. 3c). To verify that the expression of FAM83H is MYC-dependent in human cells, we induced either knock-down or overexpression of MYC in HLE and HepG2 HCC cells. The expression of mRNA and protein of FAM83H decreased with a knock-down of MYC and increased with the overexpression of MYC (Fig. 3d). Moreover, a ChIP assay demonstrated the binding of MYC to the promotor of FAM83H (Fig. 3e). More definitively, MYC-dependent transcription of FAM83H was supported by the results of a dual-luciferase reporter assay. Co-transfection of FAM83H promoter-luciferase reporter plasmid and MYC overexpression vector significantly increased luciferase activity. In contrast, co-transfection of FAM83H promoter-luciferase reporter plasmid and shRNA for MYC decreased luciferase activity (Fig. 3f).

**Figure 4:**
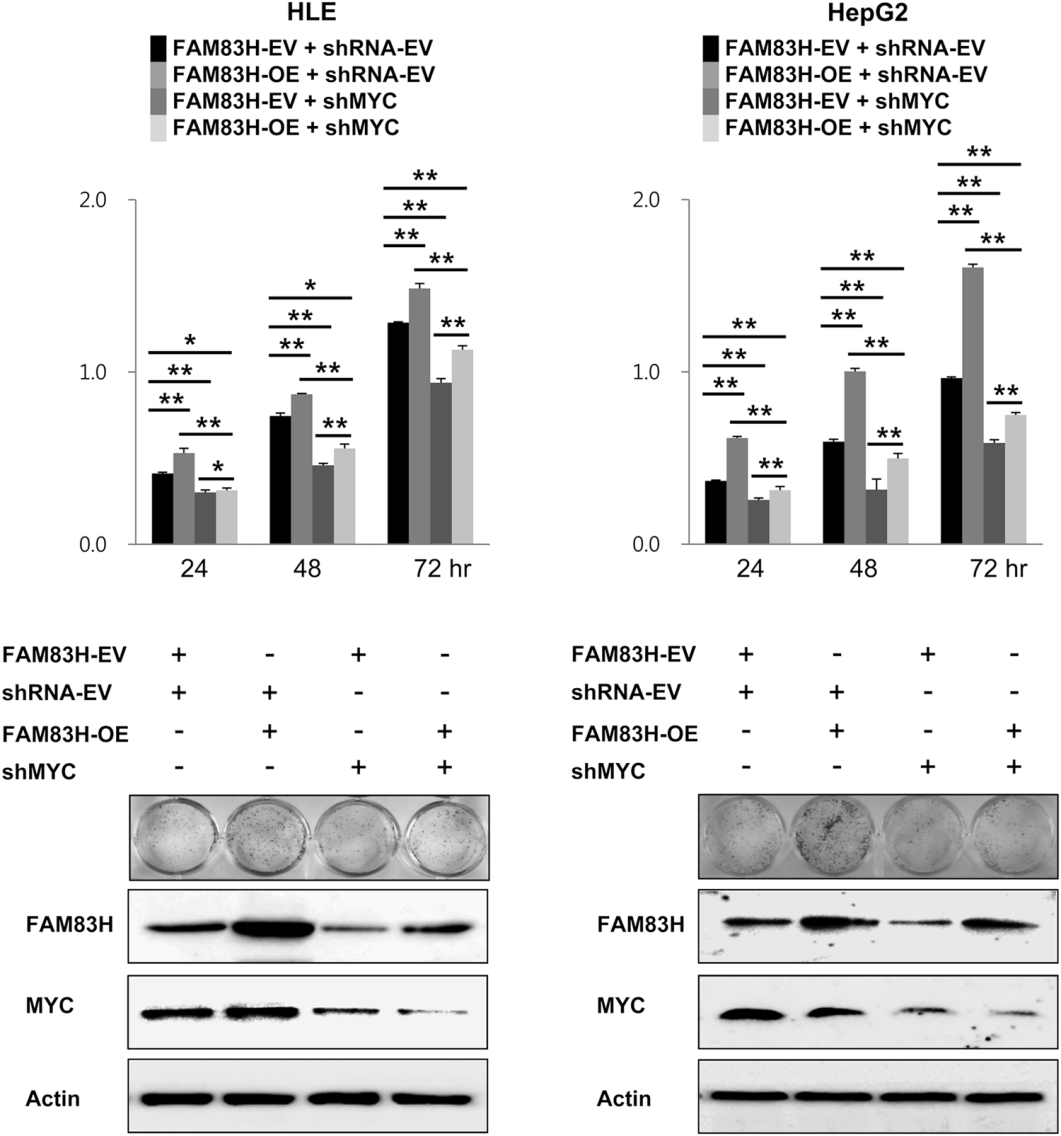
Overexpression of FAM83H increased proliferation of hepatocellular carcinoma cells with a condition of both basal expression of MYC and knock-down of MYC. Overexpression of FAM83H increased proliferation of both HLE and HepG2 cells and knock-down of MYC by shRNA for MYC decreased proliferation of both HLE and HepG2 cells as indicated by MTT and colony-forming assays. The proliferation activity of the cells co-transfected FAM83H overexpression vector and shRNA for MYC was significantly higher compared with the cells with a MYC knock-down, but still lower than cells transfected with a control vector, which have a basal expression level of MYC. OE, overexpression; the *p* values were calculated by one-way ANOVA with Tukey’s HSAD test; *p < 0.05; **p < 0.001.

In addition, we evaluated the effect of the expression of FAM83H on the proliferation of HCC cells by co-transfecting a FAM83H overexpression vector and shRNA for MYC. As we have shown in Fig. 4, the proliferative activity of HCC cells co-transfected with a FAM83H overexpression vector and shRNA for MYC was significantly lower than it was in cells transfected with a control vector, which have a basal expression level of MYC. However, FAM83H overexpression increased the proliferation in both the cells with the basal expression level of MYC, and the cells with knocked-down MYC. Therefore, these findings suggest that although the effect of FAM83H on the proliferation of HCC cells is dependent on the expression status of MYC, and that FAM83H has its own effects on the proliferation of HCC cells.

## Discussion

In this study, we investigated the role of FAM83H in liver cancer and present evidence that FAM83H might have oncogenic role. An oncogenic role for FAM83H is supported by our findings that the expression of FAM83H was relatively higher in hepatocellular carcinoma compared with normal liver tissue, and that FAM83H expression was correlated with shorter survival of HCC patients and the proliferation and invasiveness of HCC cells. In agreement with our results, gene amplification of FAM83H in liver cancer was present in 0.9% to 16.6% of cancers in a search of the cBioportal database (Supplementary Fig. S4)^22, 23^. In addition, the Oncomine database also showed higher expression of mRNA of FAM83H in liver cancer compared with normal liver tissue (Supplementary Fig. S2)^21^. The COSMIC (Catalogue of somatic mutation in cancer) database (http://cancer.sanger.ac.uk/cosmic, accession date: 28 March 2017) showed that the FAM83H gene was overexpressed in 33.5% (125 of 373) of HCCs. In line with our results, earlier DNA microarray data in HCC suggested FAM83H as a possible oncogene because an increase in FAM83H has been observed in 45.5% of cDNA data of HCC and a 2.25-fold increase in FAM83H DNA copy number has been observed in HCC^20^. Similarly, recent report has shown that FAM83 family genes are might be involved in the progression of human cancers^24^.

In our results, nuclear expression of FAM83H in HCC tissue samples was significantly associated with higher tumor stage and high preoperative serum levels of α-fetoprotein. Moreover, nuclear expression of FAM83H was an independent indicator of poor prognosis of OS and RFS of HCC patients. Although there were no previous reports specifically investigating the prognostic impact of FAM83H expression in human cancers, the public data from the cBioPortal database showed survival data according to the genetic alteration (amplification or mutation) of FAM83H in HCCs^22, 23^. One data set showed that genetic alteration of FAM83H is significantly associated with shorter disease-free survival of HCCs (Log-rank, *p* = 0.018). In another data set, although it was not statistically significant, genetic alteration of FAM83H was associated with relatively shorter OS (Log-rank, *p* = 0.108) (Supplementary Fig. S5)^22, 23^. The median survival months were 45.5 in the cases with genetic alteration of FAM83H and was 69.5 in cases without genetic alteration. However, the study for the expression of FAM83H in human cancer is very limited. Therefore, further study is needed. However, despite of this limitation, our results suggest that FAM83H affects the prognosis of HCC patients and is involved in the progression of cancer. However, how FAM83H is involved in the progression in human malignant tumors is not clear and the study of the role of FAM83H in tumorigenesis has been limited. In this study, inhibition of FAM83H *via* shRNA for FAM83H decreased the proliferation of HCC cells. In addition, the effect of FAM83H for the regulation of hepatocytes was not restricted to HCC cells. Overexpression of FAM83H increased proliferation, and knock-down of FAM83H inhibited proliferation in WRL 68 non-neoplastic hepatocyte cells (Supplementary Fig. S6). Moreover, the expression of FAM83H was involved in the regulation of G0/G1-S cell cycle progression by affecting the expression of cell cycle regulating molecules, such as P53, P27, cyclin D1, and cyclin E1. The involvement of FAM83H in the regulation of cyclin D1 expression suggests that FAM83H is associated with Wnt/β-catenin signaling because cyclin D1 is known to be down-stream in the signaling pathway of β-catenin. In addition, Wnt/β-catenin signaling is known to be critical for the regeneration of hepatocytes by regulating cellular proliferation. Supportively, the role of FAM83H for the regulation of Wnt/β-catenin signaling has been suggested in colon cancer cells. Overexpressed FAM83H decreased expression of cytoplasmic CK1α and activated β-catenin by inducing de-phosphorylation-mediated nuclear localization of it^8^. Therefore, it is possible that FAM83H-mediated change of the expression of cyclin D1 and E1 in our results might be related with the activation of TCF/LEF transcription by FAM83H/CK1α-mediated nuclear localization of β-catenin. However, further study is needed to precisely define the role of FAM83H in the regulation of cellular proliferation.

As a possible role of FAM83H in the progression of HCC, our results suggest that FAM83H might be involved in EMT. The expression of FAM83H affects the invasiveness of HCC cells and is involved in the regulation of the expression of snail and MMP2. Snail and MMP2 are important in EMT. Snail involved in EMT by repressing E-cadherin^25^ and MMP2 degrades extracellular matrix as a type IV collagenase and facilitates cancer invasion^26^. In HCC, snail^27, 28^ and MMP2^29–31^ are involved in the progression of HCC by increasing invasive and metastatic potential. Suppression of MMP2^32^ and snail^27^ inhibited the invasive and metastatic potential of HCC. Our results also support the possibility that the invasiveness of HCC is affected by the MYC-FAM83H pathway. In agreement with our results, involvement of FAM83H in the EMT of colon cancer cells has been suggested^8^. An oncogenic role of FAM83H has been suggested in colon cancer cells by inducing reorganization of actin filaments and suppressing E-cadherin expression^8^.

Another interesting finding of this study is that the expression of FAM83H is closely associated with the oncogene MYC. MYC was involved in the transcriptional regulation of FAM83H expression. In a murine Tet-O-MYC cell line, the expression of FAM83H was induced by turning on the expression of MYC, which was evidenced by qRT-PCR, western blot, and immunofluoresence staining. In addition, higher expression of FAM83H and MYC in hepatic tumor cells compared with adjacent non-neoplastic hepatocytes was demonstrated in murine Tet-O-MYC livers. Consistently, induction of MYC overexpression or knock-down of MYC in human HCC cell lines affected the expression of mRNA and protein of FAM83H. Furthermore, a ChIP assay and a dual-luciferase reporter assay evidenced that MYC transcriptionally controlled the expression of FAM83H by binding to the promotor region of FAM83H. In addition, when we evaluated the correlation of the immunohistochemical expression of FAM83H and MYC that have been reported previously in 152 HCC cases^17^, their expression patterns were significantly correlated with each other (Chi square test; *p* < 0.001 in between MYC and nuclear FAM83H expression, *p* = 0.017 in between FAM83H and cytoplasmic FAM83H expression). Immunohistochemical staining scores for nuclear and cytoplasmic FAM83H expression were also significantly higher in the MYC-positive group compared with the MYC-negative group (Supplementary Table ST2). These findings suggest that MYC-FAM83H signaling is involved in hepatic tumorigenesis and might be a potential therapeutic target for the treatment of HCC. In addition, to clearly define an independent oncogenic role of FAM83H in hepatic tumorigenesis, it might be helpful in future studies if the hepatic tumorigenic effect of FAM83H were investigated *via* the Tet-O-FAM83H transgenic mouse model.

Regarding the expression of FAM83H, it has been reported that FAM83H is mainly expressed in the cytoplasm of cells^2, 8, 10^. Intracellular expression but not extracellular matrix expression of FAM83H suggests that FAM83H could affect intracellular signaling molecules that influence the fate of cells^2, 5^. An early report demonstrated that FAM83H is expressed in the Golgi apparatus and not in the nuclei in HEK293 and HeLa cells^2^. Cytoplasmic localization of FAM83H, and its interaction with keratin filaments has been demonstrated in HCT116 and DLD1 colorectal cancer cells^10^. However, our result showed nuclear and cytoplasmic expression of FAM83H in human HCC tissue. Moreover, nuclear expression of FAM83H was more predictive of the prognosis of HCC patients than the cytoplasmic expression of FAM83H. Nuclear expression of FAM83H was an independent indicator of poor prognosis of HCC patients. Similarly, recent report has shown nuclear expression of FAM83H by immunohistochemical staining in colorectal cancer tissue and by immunofluorescence staining in RKO colorectal cancer cells^9^. Especially, nuclear localization of FAM83H was associated with poor organization of the keratin cytoskeleton, and this finding suggests that nuclear localization of FAM83H might be related with EMT of colorectal cancer cells^9^. In our results, FAM83H expression was also associated with the expression of snail and MMP2 that correlated with EMT. Therefore, although the exact mechanism by which FAM83H localization in the nuclei affects the fate of cells is not clear, these findings suggest that nuclear translocation of FAM83H from the cytoplasm might promote the progression of HCCs, just as cytoplasmic membrane-bound β-catenin activates TCF/LEF transcription factor after translocated to the nuclei. In addition, in our Kaplan-Meier survival analysis, the impact of cytoplasmic FAM83H expression on RFS was relatively less than it was on OS in the early follow-up period. These results might be associated with the difference in RFS in HCC usually found within postoperative two-years, and the survival difference found more than two-years later, which are typically associated with the difference in multi-centric carcinogenesis between the groups. However, nuclear FAM83H expression was significantly influenced for both OS and RFS. This difference may be due to the difference between the nuclear and cytoplasmic FAM83H expression in HCCs. However, despite the relatively lower prognostic impact of cytoplasmic FAM83H expression on RFS at the early follow-up period, cytoplasmic FAM83H expression was significantly associated with shorter OS (Log-rank, *p* = 0.027) and RFS (Log-rank, *p* = 0.016) at the two-year follow-up end-point. Therefore, despite some limitations of the prognostic impact of cytoplasmic FAM83H expression, our results demonstrate that cytoplasmic FAM83H expression could also be a possible prognostic indicator of HCC patients as we also have shown in multivariate analysis Model 2 in Table 2. However, further study is needed to define the role of FAM83H in tumor progression.

In contrast to FAM83H, MYC is a very well-known oncogene and the role of MYC in tumorigenesis has been extensively investigated. MYC is a transcription factor that regulates the expression of 10% to more than 15% of all cellular genes^33^. One of the main biological functions of MYC is to promote cell cycle progression^15^. Also, many studies suggested that MYC could regulate cell differentiation^34^. In general, immature proliferating cells express high level of MYC protein^35^. The role of MYC is also well understood in hepatocarcinogenesis^14^. In advanced human HCCs, chromosomal gain at the MYC locus is amongst the most prevalent reported genetic abnormalities^36^. In animal models, MYC is also reported to be a significant factor in hepatic carcinogenesis. Tumor formation was observed in mice overexpressing MYC^36^. Conversely, inactivation of MYC by a tetracycline-inducible transgenic system resulted in tumor regression with decreased proliferation, differentiation, and apoptosis of tumor cells^37^. Therefore, MYC is one of the key mediators of human hepatic carcinogenesis. Furthermore, when considering the role of FAM83H in amelogenesis, there is a possibility that MYC also might be involved in the regeneration or maintenance of teeth by engaging in amelogenesis. The possible roles of the MYC-FAM83H pathway in teeth generation is an area in which further study is needed.

In conclusion, this study demonstrated that the expression of FAM83H is transcriptionally controlled by MYC and the expression of FAM83H is associated with cellular proliferation and invasiveness. In addition, the expression of FAM83H was an independent indicator of poor prognosis of HCC patients. Therefore, our result suggests that therapeutic stratagems targeting the MYC-FAM83H signaling pathway, especially for the FAM83H-expressing poor prognostic subgroup of HCC, might be effective for the treatment of HCC patients.

## Methods

### Cell lines and animal tissue samples

The human liver cancer cell lines, HLE (mutant TP53), Huh-7 (mutant TP53) and HepG2 (wild-type TP53) were purchased from the Korean Cell Line Bank (KCLB, Seoul, Korea). The cell lines were cultured in DMEM medium supplemented with penicillin and streptomycin (100 U/ml) and 10% fetal bovine serum (Gibco BRL, Gaithersburg, MD). In addition, this study used liver tissue samples derived from bitransgenic mice that conditionally express the MYC proto-oncogene in hepatocytes. In these mice, MYC expression is activated by removing doxycycline (100 μg/ml) from the drinking water. To investigate the role of FAM83H under the influence of MYC *in vitro*, we derived tumor cell lines from liver tumors established in the Tet-O-MYC mice (Tet-O-MYC cell)^17, 19, 38^. In Tet-O-MYC cells, the addition of 5 ng/ml doxycycline prevents MYC transcription. The cell line was cultured in Dulbecco’s minimal essential medium (Supplementary Fig. S1). All animal experiments were approved by the Chonbuk National University Institutional Animal Care and Use Committee (CBNU 2016-77). All experiments were performed in accordance with relevant guidelines and regulations.

### Transfection

The MYC and FAM83H-specific shRNA expression vector was purchased from GenePharma (Shanghai, China). The MYC duplex had the sense and antisense sequences 5′-CACCGCTTGTACCTGCAGGATCTGATTCAAGAGATCAGATCCTGCAGGTACAAGCTTTTTTG-3′ and 5′-GATCCAAAAAAGCTTGTACCTGCAGGATCTGATCTCTTGAATCAGATCCTGCAGGTACAAGC-3′, respectively. The FAM83H duplex had the sense and antisense sequences 5′-CACCGCTCATCTTCAGCACGTCACATTCAAGAGATGTGACGTGCTGAAGATGAGCTTTTTTG-3′ and 5′-GATCCAAAAAAGCTCATCTTCAGCACGTCACATCTCTTGAATGTGACGTGCTGAAGATGAGC-3′, respectively. The overexpression vector for MYC (Catalog #, EX-Z2845-M03; accession #, NM_002467) and FAM83H (Catalog #, EX-Y4473-M03; accession #, NM_198488) were purchased from GeneCopoeia (Rockville, MD). JetPRIME transfection reagent (Polyplus Transfection, Illkirch, France) was used for transfection.

### Cell proliferation assay

To evaluate the proliferation of cells, a commercially available 3-(4, 5-dimethylthiazol-2-yl)-2, 5-iphenyltetrazolium bromide (MTT) cell proliferation assay (Sigma, St. Louis, MO) and a colony-forming assay were performed. The MTT assay was performed after seeding 2 × 10^3^ cells for 24, 48, and 72 h. The absorbance measured at 560 nm. The colony-forming assay was performed by seeding 1 × 10^3^ cells/well in 6-well culture plates for 10 days.

### Orthotopic tumor implantation

Orthotopic xenografts were established in 8–10-week-old male nude mice (Orient Bio. Gyonggi-Do, Korea). The HCC mice model was created by direct intrahepatic injection of 1 × 10^6^ HLE cells under anesthesia. The HLE cells were transfected with either empty vector, a FAM83H-specific shRNA expression vector, or an overexpression vector for FAM83H. Three mice were used in each experimental group. Animals were euthanized by exsanguination under sodium pentobarbital anesthesia at three weeks after transplantation of HLE cells. Tumor volume in mm^3^ was calculated by the formula: tumor volume = [(width)^2^ × length/2] mm^3^.

### Western blotting

Total proteins were lysed with PRO-PREP Protein Extraction Solution (iNtRON Biotechnology, Korea) containing 1x phosphatase inhibitor cocktails 2, 3 (Sigma) and probed with primary antibodies for FAM83H (Bethyl Laboratories, Montgomery, TX), MYC (Abcam, Cambridge, UK), P53 (Novocastra, Newcastle, UK), P27 (Santa Cruz Biotechnology, Santa Cruz, CA), cyclin D1 (Santa Cruz Biotechnology), cyclin E1 (Abcam), MMP-2 (Santa Cruz Biotechnology), Snail (Abcam), E-cadherin (Cell Signaling Technology, Beverly, MA), and actin (Sigma).

### Chromatin immunoprecipitation assay

The chromatin immunoprecipitation (ChIP) kit was purchased from Cell Signaling Technology (number 9002, Cell Signaling Technology). ChIP was performed according to the manufacturer’s instructions with HLE cells. Formaldehyde was added at a final concentration of 1% directly to media containing the HLE cells. Fixation proceeded at room temperature for 10 min and was stopped by the addition of glycine to a final concentration of 0.125 M for 15 min. Cells were centrifuged and rinsed 3 times in cold PBS with 1 mM PMSF. Then, cell nuclei were collected according to the manufacturer’s protocol. The samples were sonicated on ice with a sonicator at setting 5 for six 10 s pulses to an average chromatin length of approximately 300 to 800 bp. For the immunoprecipitation, antibodies for MYC (Abcam), histone H3 (positive control, Cell Signaling Technology), and normal rabbit IgG (negative control, Cell Signaling Technology) were added in combination to the nuclear sonicate. Then the immunoprecipitation was eluted and the DNA was recovered. DNA obtained from immunoprecipitation samples were amplified by conventional PCR using primers to detect FAM83H promoter gene locus and ribosomal protein L30 (RPL30) (Cell Signaling Technology) gene locus.

### Dual-luciferase assay

HLE cells were grown in a 6-well plate and transfected with either a control vector (1 μg), shRNA for MYC (1 μg) or a MYC overexpression vector (1 μg). Each well was co-transfected with Renilla luciferase expression vector and FAM83H promoter-luciferase reporter plasmid (Switchgear Genomics, Menlo Park, CA). At 48 h after transfection, the intracellular hRluc and firefly luciferase activities were assayed using a dual-luciferase reporter assay system (Promega, Madison, WI) according to the manufacturer’s instructions. The ratio of Firefly/Renilla activity was calculated.

### Immunofluorescence staining

To evaluate the localization of FAM83H and MYC, immunofluorescence staining was performed. The HLE and HepG2 cells were fixed with methanol and incubated with anti-FAM83H (1:100, Bethyl Laboratories) and anti-MYC (1:100, Abcam) antibodies and then incubated with Alexa FluorVR 488 anti-rabbit IgG or Alexa FluorVR 594 anti-mouse IgG (Invitrogen, Carlsbad, CA). The slides were counterstained with DAPI, and the images were acquired using a Nikon ECLIPSE E600 fluorescence microscope (Nikon, Japan) and software (Nikon ACT-1 2.62, Nikon).

### *In vitro* migration and invasion assays

The migration assay was performed using a 24-transwell migration chamber with 8 μm-pore sized filters (BD Biosciences, San Jose, CA). 1 × 10^5^ cells were seeded in the migration chamber in which the bottom chamber consisted of 10% FBS. For the invasion assay, 2 × 10^5^ cells were seeded in the upper chamber containing an 8 μm-pore sized Matrigel Invasion Chamber (BD Biosciences). DMEM with 10% FBS was added in the lower chamber in the invasion assay. The migration and invasion chambers were incubated at 37 °C for 24 hours. Thereafter, the lower surface of the filter was stained with DIFF-Quik staining solutions (Sysmex, Japan), and the number of cells which had migrated or invaded the filter were counted in five microscopic fields (magnification ×200) per well.

### Quantitative reverse-transcription polymerase chain reaction

RNA isolation was performed with the RNeasy Mini Kit (Qiagen Sciences, Valencia, CA). After DNase treatment, reverse transcription of 1.5 μg RNA was performed with Taqman Reverse Transcription Reagents (Applied Biosystems, Foster City, CA). Quantitative reverse-transcription polymerase chain reaction was carried out using the Applied Biosystems Prism 7900HT Sequence Detection System and Sybr Green polymerase chain reaction Master Mix (Applied Biosystems). All experiments were performed in triplicate, and the results were normalized to the expression of the glyceraldehyde-3-phosphate dehydrogenase reference housekeeping gene. Primer sequences for quantitative reverse-transcription polymerase chain reaction are listed in Supplementary Table ST1.

### Cell cycle analysis

Transfected HLE and HepG2 cells were harvested, washed with PBS and fixed in 70% ethanol at 4 °C overnight. Fixed cells were incubated with PBS containing 50 μg/mL propidium iodide (Sigma) and 50 μg/mL RNase A (Sigma) at 37 °C for 30 min. Thereafter, DNA content was measured by a FACStar flow cytometer (Becton-Dickinson, San Jose, CA) and analyzed using FlowJo software (Tree Star, Ashland, OR).

### Hepatocellular carcinoma patients and tissue samples

HCC patients who underwent radical resection in Chonbuk National University Hospital between January 1998 and August 2009 were considered for this study. Thereafter, 163 cases of HCCs that the medical records, the histologic slides, and paraffin tissue-blocks were available were included in this study. The median follow-up duration was 61 months (range: 1–175). Among the 163 HCC patients, 113 patients experienced relapse and 83 patients died from HCC by the follow-up endpoint. The five- and ten-year survival rates for the HCC patients were 62% and 43%, respectively. All the cases were reviewed and classified by two pathologists (JKY and NSJ) according to the criteria of the World Health Organization Classification^39^. Tumor stage was assigned according to the staging system of the American Joint Committee on Cancer^40^. Three normal liver tissue samples from autopsy were obtained from the Department of Forensic Medicine of Chonbuk National University Medical School. This study obtained institutional review board approval from Chonbuk National University Hospital (IRB number, CUH 2014-05-038-001 and CUH 2016-09-004-002) and written informed consents were obtained from the patients or their legal guardian. All experiments were performed in accordance with relevant guidelines and regulations.

### Immunohistochemical staining and scoring for tissue microarrays

Immunohistochemical staining was performed in paraffin-embedded tissue blocks from the tissue microarray established from human HCC tissue and mouse tissue. The tissue microarray for human HCC was performed on one 3.0 mm core per HCC case from the most representative intact area with the highest histologic grade. The tissue sections were boiled in Dako Target Retrieval Solution (pH 6.0, DAKO, Glostrup, Denmark) in a microwave oven for 12 minutes for antigen retrieval. Thereafter, primary antibodies for FAM83H (1:100, Bethyl Laboratories), and MYC (1:100, Abcam) were used for immunohistochemical detection. Scoring of immunohistochemical staining for FAM83H in human HCC samples was performed separately for the nuclear and cytoplasmic expression of FAM83H in tumor cells by two pathologists (KYJ and KMK) by consensus without knowledge of the clinicopathological information under a multi-viewing microscope. Nuclear and cytoplasmic expression of FAM83H in tissue microarray blocks were evaluated by the sum of the staining intensity scores and the staining area scores. The staining intensity score ranged from zero to three (0, no staining; 1, weak staining; 2, intermediate staining; 3, strong staining). The staining area score was ranged from zero to five (0, no stained cells; 1, 1% of the cells stained positive; 2, 2–10% of the cells stained positive; 3, 11–33% of the cells stained positive; 4, 34–66% of the cells stained positive; 5, 67–100% of the cells stained positive)^41–43^. The sum score ranged from zero to eight. Subsequently, sum scores of the nuclear and cytoplasmic expression of FAM83H were grouped as positive or negative by receiver operating characteristic curve analysis at the highest positive likelihood ratio point for the estimation of death of HCC patients^42, 43^.

### Statistical analysis

The survival of HCC patients was evaluated by analysis of the OS and RFS. The follow-up end point was the date of last contact or death through June 2013. OS was calculated from the date of diagnosis to the date of death by HCC or last contact. Patients who were alive at last contact or died from other causes were treated as censored for OS analysis. RFS was calculated from the date of diagnosis to the date of relapse of any type, death from HCC, or last contact. Patients who were alive at last contact without relapse of the tumor or who died from other causes were treated as censored for RFS analysis. The prognostic impact of clinicopathologic factors and expression of each marker on OS and RFS was evaluated by univariate and multivariate Cox proportional hazards regression analyses. Kaplan-Meier survival analysis was performed and a survival curve generated to illustrate the impacts on OS and RFS. Pearson’s chi-square test, Student’s *t*-test, and the one-way ANOVA with Tukey’s HSD test were used to compare the values between the groups. All experiments were performed in triplicate, and representative data are presented. Statistical analysis was performed using SPSS software (IBM, version 19.0, CA). The *p* values less than 0.05 were considered statistically significant.

## Electronic supplementary material

Supplementary information
