# Supplementary material for: FAM83H is involved in the progression of hepatocellular carcinoma and is regulated by MYC
Source: Sci Rep. 2017 Jun 12;7:3274. doi: 10.1038/s41598-017-03639-3 (PMC5468291; doi:10.1038/s41598-017-03639-3)
Supplement: Supplementary file 1 — Supplementary information [file 41598_2017_3639_MOESM1_ESM.pdf]

Supplementary information

FAM83H is involved in the progression of hepatocellular carcinoma and is regulated by MYC

Kyoung Min Kim[1], See-Hyoung Park[2], Jun Sang Bae[1], Sang Jae Noh[3], Guo-Zhong Tao[4], Jung Ryul Kim[5], Keun Sang Kwon[6], Ho Sung Park[1], Byung-Hyun Park[7], Ho Lee[3], Myoung Ja Chung[1], Woo Sung Moon[1], Karl G Sylvester[4] & Kyu Yun Jang[1,*]

Department of [1]Pathology, [3]Forensic Medicine, [5]Orthopedic Surgery, [6]Preventive Medicine, and [7]Biochemistry, Chonbuk National University Medical School, Research Institute of Clinical Medicine of Chonbuk National University-Biomedical Research Institute of Chonbuk National University Hospital and Research Institute for Endocrine Sciences, Jeonju, Republic of Korea.

[2]Department of Bio and Chemical Engineering, Hongik University, Sejong, Republic of Korea.

[4]Department of Surgery, Division of Pediatric Surgery, Stanford University School of Medicine, Stanford, California, USA.

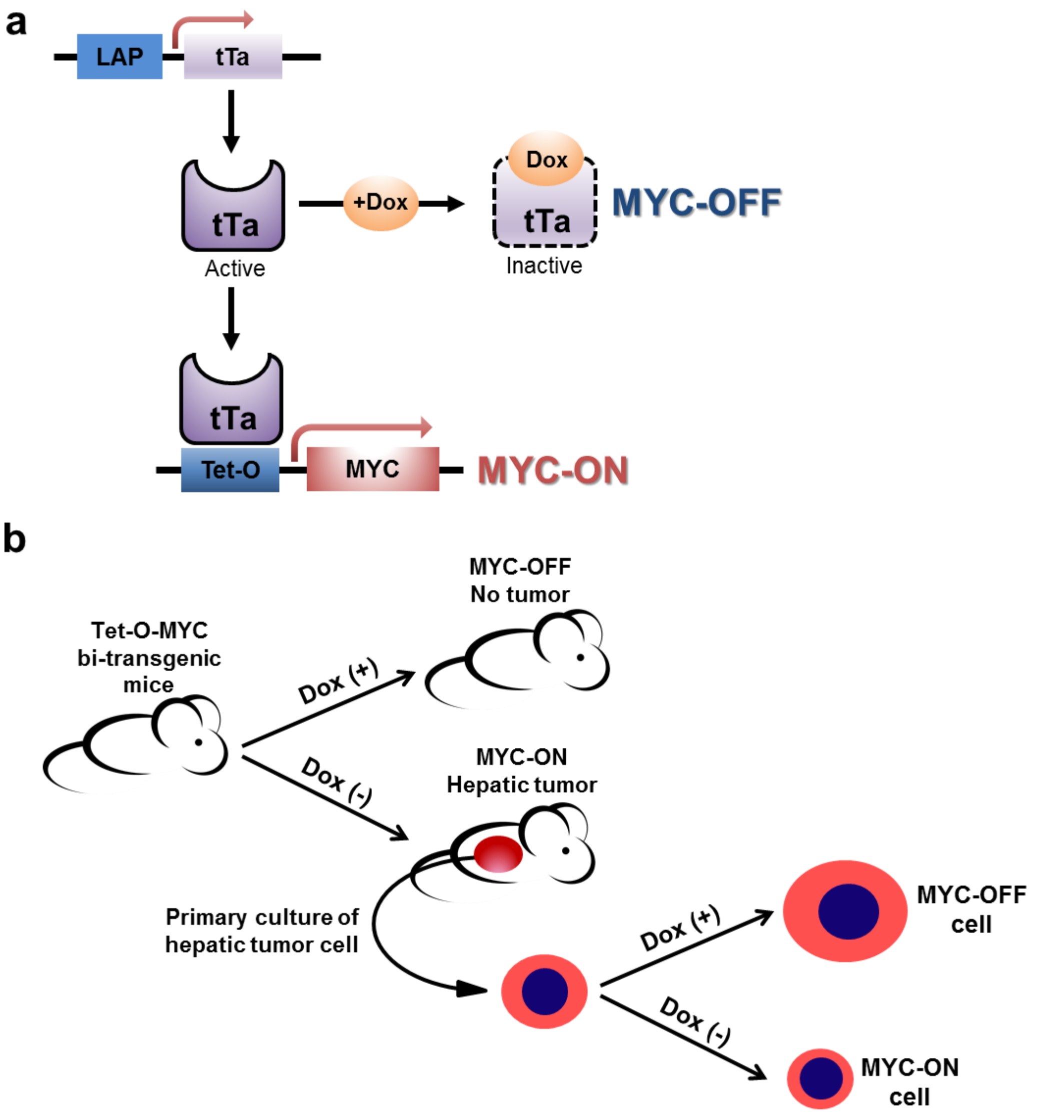

Supplementary Figure S1. Biological characteristics of a murine hepatic cell line conditionally overexpressing MYC. (a) Conditional overexpression of MYC in hepatocytes under the control of the tetracycline analog doxycycline was achieved by engineering mice with liver associated protein (LAP) and tetracycline-response promoter (Tet-O). (b) The hepatic cell line was established from the hepatic primary tumor developed in bi-transgenic mice (Tet-O-MYC mice). In basal culture conditions, the cells overexpress MYC (MYC-ON cells) and MYC transcription is turned off (MYC-OFF cells) *via* addition of 5 nM doxycycline.

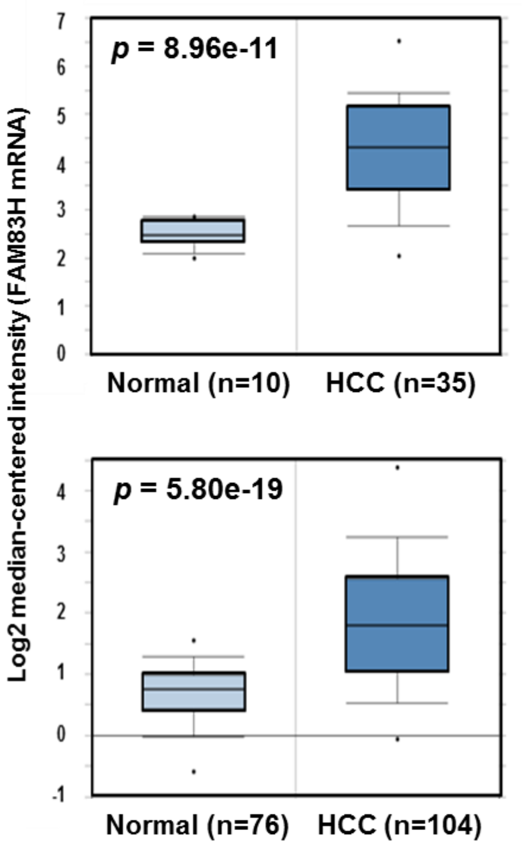

Supplementary Figure S2. The mRNA expression of FAM83H in hepatocellular carcinoma. The expression of mRNA of FAM83H was significantly increased in hepatocellular carcinoma compared with normal liver tissue as determined *via* the Oncomine database (https://www.oncomine.org, accession date: 11 January 2017).

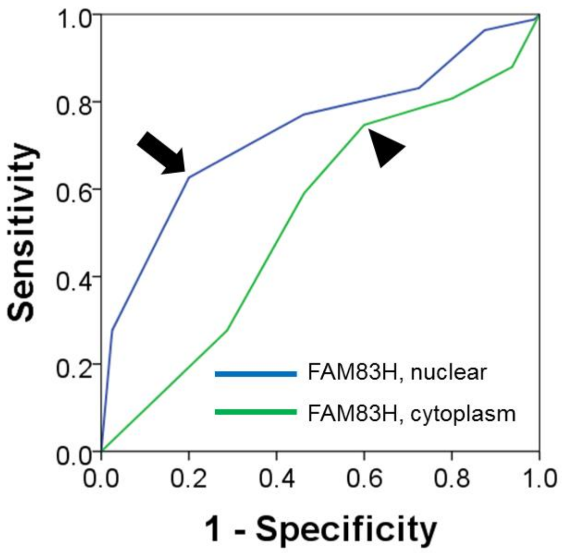

	Cut-Off	AUC	*p*
FAM83H, nuclear	All	0.733	< 0.001
	≥ 7	0.713	< 0.001
FAM83H, cytoplasm	All	0.538	0.405
	≥ 6	0.573	0.105

Supplementary Figure S3. Statistical analysis to determine cut-off points for the nuclear and cytoplasmic expression of FAM83H in human hepatocellular carcinomas. To determine the cut-off points for the immunostaining for FAM83H, receiver operator characteristic curves analysis was performed. The cut-off points were determined at the highest sensitive and specific points to estimate death of hepatocellular carcinoma patients. The arrow and arrow head indicate the cut-off points for the nuclear and cytoplasmic expression of FAM83H, respectively.

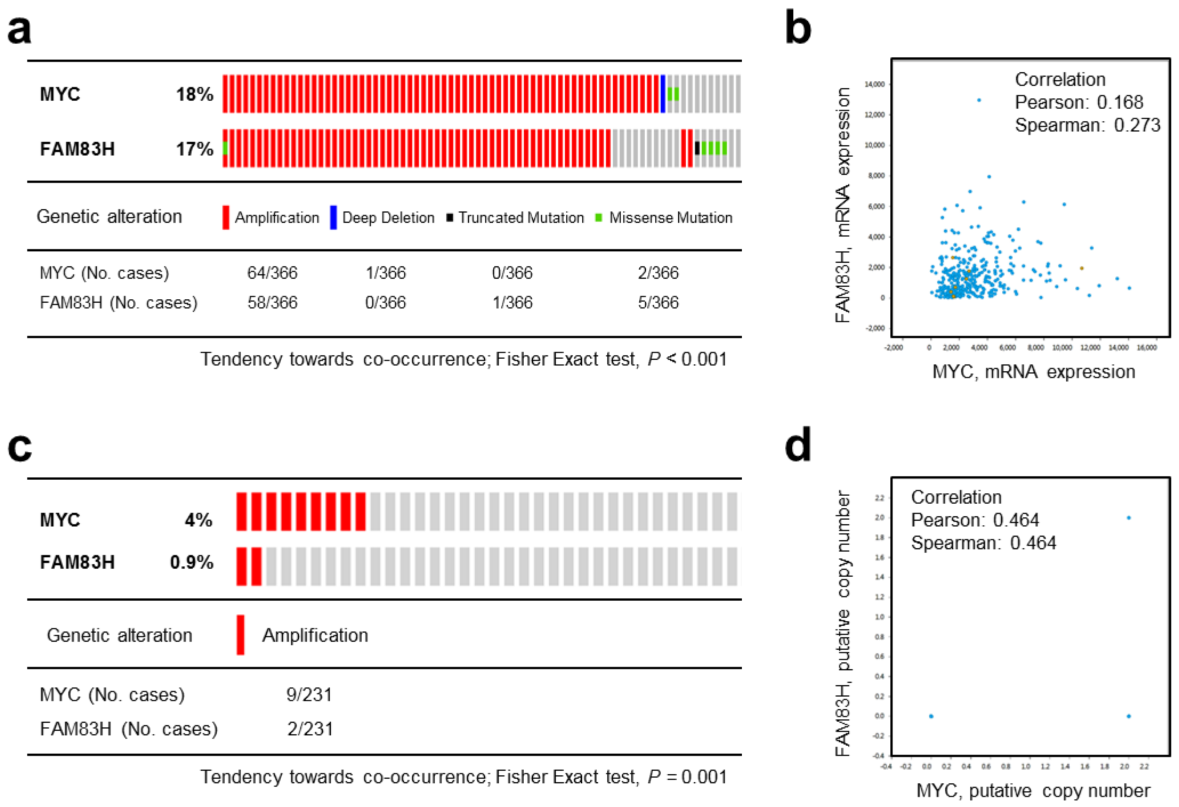

Supplementary Figure S4. The association between genetic alterations of MYC and FAM83H in human hepatocellular carcinomas. A search of the cBioPortal database (http://www.cbioportal.org, accession date: 11 January 2017) showed a significant correlation between the genetic alteration of FAM83H and MYC genes. (a,b) One data set showed co-occurrence of a genetic change of FAM83H and MYC genes (Fisher Exact test; $p < 0.001$, Pearson correlation; $p = 0.168$, Spearman correlation; $p = 0.273$). Another data set also showed co-occurrence of genetic change of FAM83H and MYC genes (Fisher Exact test; $p = 0.001$, Pearson correlation; $p = 0.464$, Spearman correlation; $p = 0.464$).

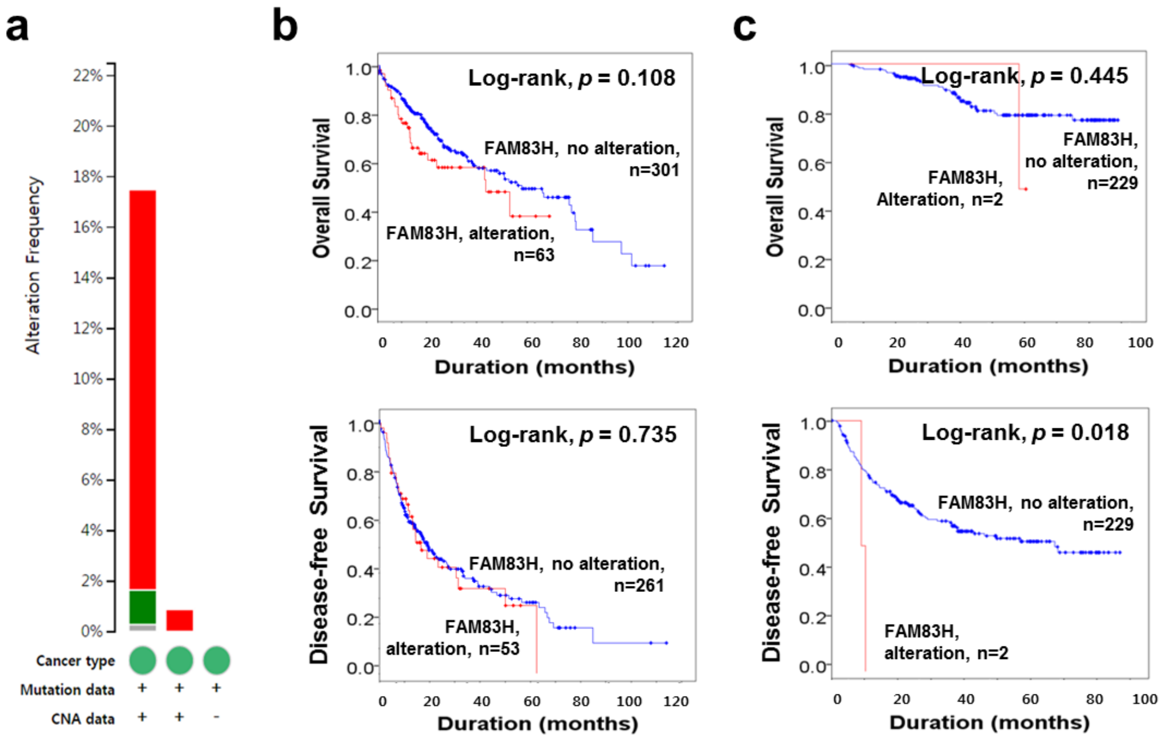

Supplementary Figure S5. Genetic alteration of FAM83H in human hepatocellular carcinomas. (a) A search of the cBioPortal database (http://www.cbioportal.org, accession date: 11 January 2017) showed that two of three data sets have the genetic alteration (amplification or mutation) of FAM83H in liver cancer. (b,c) Kaplan-Meier survival curve according to the genetic alteration of FAM83H in hepatocellular carcinomas.

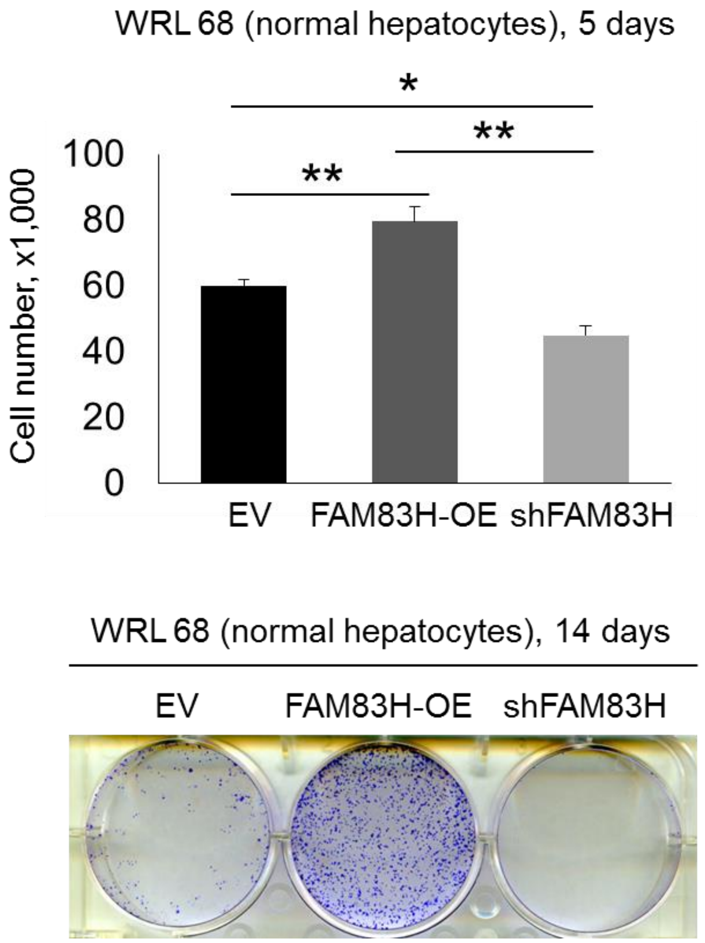

Supplementary Figure S6. The expression of FAM83H is associated with the proliferation of WRL 68 non-neoplastic hepatocytes. The knock-down of FAM83H significantly inhibited the proliferation, and overexpression of FAM83H (induced by transfection of FAM83H) increased proliferation of WRL 68 non-neoplastic hepatocytes as indicated with cell counting and colony-forming assays. The WRL 68 cells were purchase from ATCC (ATCC CL-48, Manassas, VA, USA). EV, empty vector; OE, overexpression; the *p* values were calculated by one-way ANOVA with Tukey's HSAD test; *, $p < 0.05$; **, $p < 0.001$.

Supplementary Table 1. The primer sequences used for the quantitative real-time polymerase chain reaction

Gene		Primer sequence	Product size	Accession number
FAM83H	forward	CATGGTCCAGACAACCTGTG	214	NM_198488.3
	reverse	GCTGGATACCAGGAGGACAA		
MYC	forward	TTCGGGTAGTGGAAAACCAG	203	NM_002467.4
	reverse	CAGCAGCTCGAATTTCTTCC		
TP53 (p53)	forward	GGCCCACTTCACCGTACTAA	156	NM_001276760.1
	reverse	GTGGTTTCAAGGCCAGATGT		
CDKN1B (p27)	forward	AGATGTCAAACGTGCGAGTG	154	NM_004064.4
	reverse	TCTCTGCAGTGCTTCTCCAA		
CCND1 (Cyclin D1)	forward	GAGGAAGAGGAGGAGGAGGA	236	NM_053056.2
	reverse	GAGATGGAAGGGGGAAAGAG		
CCNE1 (Cyclin E1)	forward	AGCGGTAAGAAGCAGAGCAG	189	NM_001238.3
	reverse	TTTGATGCCATCCACAGAAA		
SNAL1 (Snail)	forward	ACCCCACATCCTTCTCACTG	217	NM_005985.3
	reverse	TACAAAAACCCACGCAGACA		
MMP2	forward	ATGACAGCTGCACCACTGAG	174	NM_004530.5
	reverse	ATTTGTTGCCCAGGAAAGTG		
GAPDH	forward	AACAGCGACACCCACTCCTC	258	NM_001256799.1
	reverse	GGAGGGGAGATTCAGTGTGGT		
FAM83H, mouse	forward	CCCACTTCAACCTACCCAGA	221	NM_001168253.1
	reverse	CTCTCATTCTTGGGGCTCAG		
MYC, mouse	forward	GCCCAGTGAGGATATCTGGA	226	NM_001177352.1
	reverse	ATCGCAGATGAAGCTCTGGT		
GAPDH, mouse	forward	AACTTTGGCATTGTGGAAGG	223	NM_001289726.1
	reverse	ACACATTGGGGGTAGGAACA		

Supplementary Table 2. Correlation between the immunohistochemical expressions of FAM83H and MYC in 152 cases of hepatocellular carcinomas

Characteristics		No.	MYC		
			Negative	Positive	*p*
Pearson Chi square test					
FAM83H, nuclear	Negative	89	59 (66%)	30 (34%)	< 0.001
	Positive	63	22 (35%)	41 (65%)	
FAM83H, cytoplasmic	Negative	49	33 (67%)	16 (33%)	0.017
	Positive	103	48 (47%)	55 (53%)	
t-test					
FAM83H, nuclear	IHC score (mean ± SD)	152	5.4 ± 1.5	6.4 ± 1.6	< 0.001
FAM83H, cytoplasmic	IHC score (mean ± SD)	152	11.3 ± 2.6	12.9 ± 2.8	< 0.001

IHC, immunohistochemical staining; SD, standard deviation
